# Electroanalytical Strategies for Local pH Sensing at Solid–Liquid Interfaces and Biointerfaces

**DOI:** 10.1021/acssensors.4c01391

**Published:** 2024-09-04

**Authors:** Isabell Wachta, Kannan Balasubramanian

**Affiliations:** Department of Chemistry and School of Analytical Sciences Adlershof (SALSA), Humboldt-Universität zu Berlin, 10099 Berlin, Germany

**Keywords:** pH sensor, ion selective electrode, ultramicroelectrode, scanning electrochemical microscopy, potentiometry, voltammetry, electrocatalysis, corrosion, extracellular pH, electrified interface

## Abstract

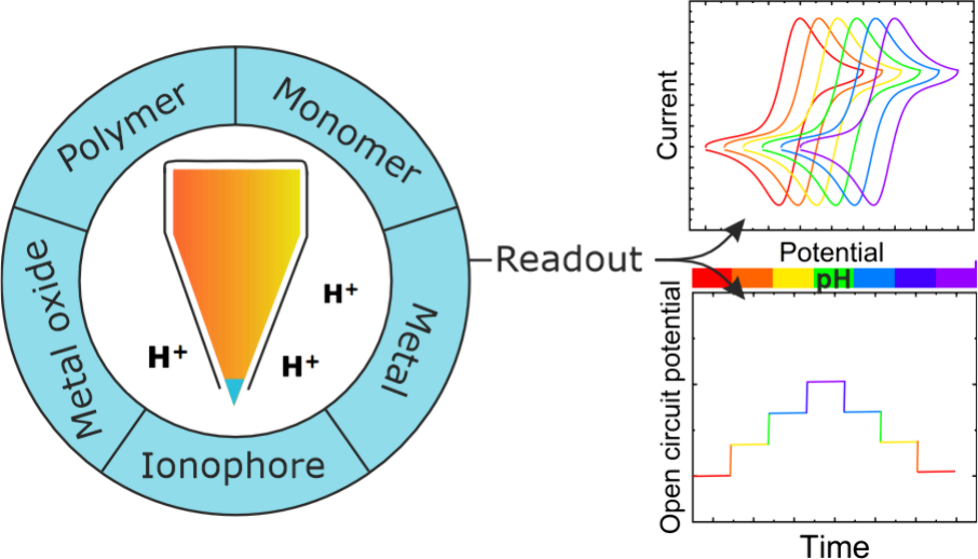

Obtaining analytical information about chemical species at interfaces is fundamentally important to improving our understanding of chemical reactions and biological processes. pH at solid–liquid interfaces is found to deviate from the bulk solution value, for example, in electrocatalytic reactions at surfaces or during the corrosion of metals. Also, in the vicinity of living cells, metabolic reactions or cellular responses cause changes in pH at the extracellular interface. In this review, we collect recent progress in the development of sensors with the capability to detect pH at or close to solid–liquid and bio interfaces, with spatial and time resolution. After the two main principles of pH detection are presented, the different classes of molecules and materials that are used as active components in these sensors are described. The review then focuses on the reported electroanalytical techniques for local pH sensing. As application examples, we discuss model studies that exploit local pH sensing in the area of electrocatalysis, corrosion, and cellular interfaces. We conclude with a discussion of key challenges for wider use of this analytical approach, which shows promise to improve the mechanistic understanding of reactions and processes at realistic interfaces.

The pH value^1^ is one of, if not the most frequently measured parameter in laboratories. Knowing the pH value in a chemical system is essential, since it often dictates the kinetics and mechanisms of several chemical reactions and processes.^2^ In heterogeneous reactions, the concentration of chemical species at the solid–liquid interface varies significantly from the bulk. For example, in electrocatalysis, there is recent evidence for drastic variations in pH at the catalyst–electrolyte interface even when working in buffers.^3−6^ In biological systems, it is known that cellular metabolism and activity affect the local pH, e.g., in the vicinity of cancer cells.^7^ Another process, where pH changes locally, is during the corrosion of bulk metals or dissolution of nanoparticles.^8^ Even if protons are not directly involved in a reaction, the local change in pH may affect reaction rates, e.g., in enzyme kinetics. Moreover, the changes are expected to play a crucial role in the underlying mechanisms of the involved processes or reactions. Hence, there is a growing need to understand the evolution of local pH during the course of heterogeneous reactions or phenomena at solid–liquid or cellular interfaces. Local pH information can be gathered by using optical or electrochemical transduction methods.

Optical detection constitutes a simple route to measure local pH^9^ e.g. by exploiting the pH-sensitive fluorescence of dyes such as (carboxyl)fluorescein.^10−14^ The fluorophore is just added to the medium^12,13^ or immobilized on an optical fiber^10^ and changes in pH can be monitored by focusing on a chosen detection volume close to the interface of interest. In order to improve the pH-resolution and the detection range, ratiometric probes have also been designed.^10,15−18^ Apart from fluorescence,^19^ Raman^6,20,21^ and IR spectroscopy^3,22^ have been utilized to monitor local pH. Often, the used probes are directly affected by the pH, e.g. a change in the protonation state, which modulates the vibrational intensity.^21^ Alternatively, the relative vibrational intensity of a weak-acid/conjugate base pair (e.g., CO_2_/HCO_3–_)^3,22^ is recorded from which the local pH is indirectly extracted. In pure perchlorate solutions, a different approach has been taken, exploiting the prominent vibrational signature of the dissociated ClO_4_^–^ ion.^20^ Here, electroneutrality is assumed in the volume of the optical probe (spot diameter of around 3 μm). As a result, the proton concentration is assumed to be equal to the concentration of the perchlorate ions. By measuring the Raman signal, which is proportional to [ClO_4_^–^], the pH is estimated quantitatively. Since these methods mostly rely on measuring the optical intensity or absorption, calibration is difficult, since the pH-dependence is nonlinear. For direct pH detection, the usable pH range is limited by the acid dissociation constant (p*K*_a_) of the probe.^11,15,23^

Although optical detection has the advantages of easy miniaturization and analysis in the optical far-field in a noncontact manner,^24^ there are a few drawbacks. The spatial resolution is often restricted by the diffraction limit, and the samples in some cases have to be transparent or thin. Moreover, the attainable pH resolution is typically limited to around 0.1 pH units. In many cases, the amount of optical probe added is quite low. However, since this is a marker-based strategy, the presence of the marker itself may in some cases additionally affect the local pH. Finally, the measurements have to be often carried out under laser illumination, which may induce additional unwanted photochemical reaction pathways or will present a severe limitation in time resolution or analysis time in order to minimize local heating. Despite these drawbacks, there are several examples of corrosion,^10,11,25^ electrocatalysis,^3,12,13,22^ and extracellular fluorescence labeling,^26^ where it has been possible to monitor local pH variations optically. A very elaborate review on this topic is also available.^27^

Electrochemical methods for local pH detection have demonstrated the capability to overcome several of the above-mentioned disadvantages. For example, the spatial resolution is limited mainly by the size of the electrode, which can be in the nanometer range. In this review, we will first focus on the general principles for detecting pH and consider the hurdles to be overcome toward realizing miniature pH sensors. These principles are based on a reaction equilibrium involving protons. Subsequently, we discuss electroanalytical strategies to interrogate this reaction equilibrium in order to decipher the local pH. Several material and molecular systems have been reported to realize electroanalytical miniaturized pH sensors, which will be presented next. Following this, selected examples of the application of local pH sensing in the area of electrocatalysis, corrosion science, and biointerfaces will be outlined. Based on this survey, the challenges facing this analytical discipline will be finally critically analyzed.

## General Principles of Local pH Detection

There are two fundamental principles for the detection of pH using electroanalytical methods. The first category is based on the measurement of the potential developing across a membrane, which is in equilibrium with protons in solution. The glass electrode^28^ of a typical pH meter works using this principle. The second category involves the interrogation of an immobilized redox active species/layer, whose formal potential is controlled by solution pH.

### Detection Based on Membrane Potential

**Figure 1:**
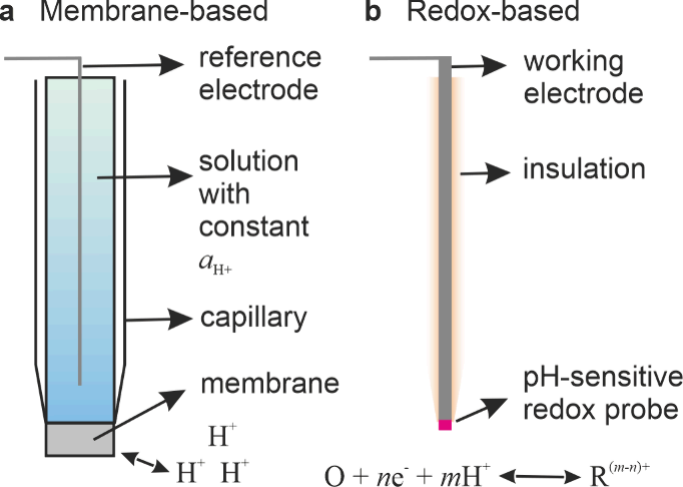
Typical layouts for (a) a membrane-based pH electrode consisting of a reference electrode and a membrane that separates the inner solution from the analysis solution, where the H^+^ activity needs to be measured, and (b) a redox-based pH electrode consisting of a working electrode modified with a pH-sensitive electroactive species at the end and otherwise passivated with an insulating layer.

The membrane potential is the standard transduction strategy used widely for measuring the pH of solutions. Figure 1(a) shows a scheme of the buildup of such a sensor. It comprises a membrane, which has the capability of proton exchange with the solution on both sides. An imbalance in the activity of protons (*a*_H^+^_) leads to a nonzero membrane potential. In order to measure this potential, the membrane is embedded in a capillary filled with a standard solution whose *a*_H^+^_ is maintained constant (*a*_*H*+_^*inner*^). When such a capillary is immersed in the sample test solution, a potential develops across the membrane depending on the difference in *a*_H^+^_ with respect to that of the inner solution. In the case of the glass electrode, this membrane is composed mainly of SiO_2_ in addition to other oxides.^29^ The key equilibrium between the surface oxide groups and H^+^ is as follows:
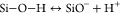
1This reaction does not involve any change in the oxidation states. The membrane potential developed in this case is a linear function of pH, given by the Nernst equation as
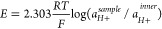
2The potential can be measured by using two reference electrodes, one placed within the capillary and the other placed in the test solution. The electroanalytical technique usable here is potentiometry. Upon proper calibration,^30^ the membrane potential is found to be directly proportional to the pH of the test solution. The range of usable pH is limited by the membrane stability as well as other dopants in glass, which interfere with the above equilibrium.^30−32^

Local pH sensing requires electrodes with micro- to nanosized diameters. The structure of a conventional glass pH electrode includes a solution inside of the device. Its high resistance and fragility make it very unfavorable for direct miniaturization.^33−35^ In order to overcome this drawback, often a polymer membrane doped with ionophores is used, which provides a good sensitivity in a broad pH range.^33,36^ Hydrogen ionophores or proton ionophores are able to undergo a complexation equilibrium selectively with protons.^24^ A new concept based on the measurement of the ionic conductivity of the membrane fixed in a nanopore has also been reported.^37^ The membrane in this case consisted of glucose oxidase molecules embedded in a poly(lysine) matrix and showed pH-dependent ionic conductivity. This dependence was attributed to a preferential permeability for cations at high pH and anions at low pH due to the rapid protonation/deprotonation of the membrane from protons in solution.

A variant of membrane-based detection is the so-called solid contact ion selective electrodes (contact-ISE). In such electrodes, the internal solution/membrane interface is replaced by a contact with a solid electrode. In this format, the solid electrode directly detects the protonation equilibrium of the coated membrane. One fundamental challenge with this type of sensors is the necessity to realize an appropriate ion-to-electron transduction interface between the solid and the membrane.^35^ Nanomaterials such as carbon nanotubes, graphene derivatives, gold and silver nanoparticles and CdS/CdSe quantum dots have been proposed as a solution for this purpose.^38^ One disadvantage is that the potential stability is very low. Although such electrodes are widely used for detecting a range of ions, e.g. K^+^, Ca^2+^, Ag^+^, and ClO_4_®,^38−40^ it is much less common^36,41^ for detecting pH locally at interfaces.

### Detection Based on pH-Dependent Formal Potential

A second strategy for realizing pH sensors involves the immobilization of a redox active molecule or the direct fabrication of a redox material on an electrode. In both cases, the formal potential of the redox species varies as a function of pH. In other words, an electron transfer reaction is coupled with protons as reactants or products. A very basic scheme of this methodology is shown in Figure 1(b). Here the redox probe is immobilized, deposited, or polymerized at the end of an otherwise insulated electrode. The formal potential can be read out using different electroanalytical strategies, potentiometry or voltammetry. Miniaturization is rather straightforward here, since it is mainly necessary to realize conducting electrodes in the micro/nanoscale regime. Here, ultramicroelectrodes (UMEs)^42,43^ or nanoelectrodes (NEs)^44,45^ are ideally suited for local pH sensing, where it is sufficient to work in a two-electrode configuration.^46^ The counter electrode is often a simple Ag/AgCl reference.

Consider a general redox reaction between an oxidized (**O**) and reduced (**R**) species involving *m* protons and *n* electrons:
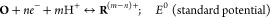
3Assuming we have immobilized **O**/**R** species on the electrode surface (activity = 1), the formal potential (*E*^0^′) using the Nernst equation can be derived as
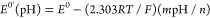
4with *R*, *T*, and *F* denoting the gas constant, temperature, and the Faraday constant, respectively. At room temperature (*T* = 298 K) this equation simplifies to
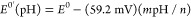
5Thus, the formal potential is found to vary linearly with a slope of (59.2 mV × *m*/*n*) as a function of pH. Often *m* = *n* in the case of metal oxides and redox active probes, where a theoretical maximum slope of 59.2 mV/pH can be observed, referred to as the Nernstian limit. However, due to experimental restrictions, the observed slope values can be below this theoretical limit.

In order that we reliably read out the formal potential, it is necessary that the kinetics of the reaction is fast and the exchange current density is high.^47^ Sluggish kinetics may limit the responsivity, especially when the measurements are carried out by using potentiometry. Moreover, the formal potential of the redox active species varies as a function of ionic strength.^47^ Typically, the pH dependence of the formal potential is calibrated by using standard solutions. For a measured calibration to be valid, the dependence on the ionic strength must be considered. The ionic strengths of these standards can be chosen to match the situation in which the desired experiment needs to be carried out.

It is also important that the probe is well-attached to the electrode surface. A fundamental problem in immobilized electrodes is the leaching of the redox probe, which may lead to a gradual loss in the signal-to-noise ratio. Analogous to optical pH sensors, the usable pH range is dictated by the p*K*_a_ of the redox species. This is because, in equation 3, the formal potential of the redox reaction varies depending on the protonation state of the redox active species. Moreover, the number of protons involved in the reaction may vary depending on the protonation state, e.g., in the case of methylene blue.^48^ In this case, the formal potential exhibits more than one slope in different pH regimes. By using voltammetric techniques, it is in principle possible to attain a better time resolution and a smaller response time in comparison with a potentiometric readout.^49,50^ Moreover, the spatial resolution can be expected to be superior due to the use of UMEs or NEs.

## Molecules and Material Systems for Local pH Sensing

Several candidate probes have been used for pH sensing, which can be grouped as follows: ionophores, metals, metal oxides, and monomeric or polymeric redox active molecules. The ionophores are mainly used in the membrane-based transduction strategy. Metal oxides are used predominantly in contact-ISE based systems. In the case of the other three candidates, metals, monomers, and polymers, the pH-dependent formal potential variation is exploited to build modified electrodes for local pH sensing. In this section, we take a closer look at these different molecular and material systems. The presented classes of active layers are used for voltammetric or potentiometric pH sensors depending on the sensing mechanism.

### Ionophores

**Figure 2:**
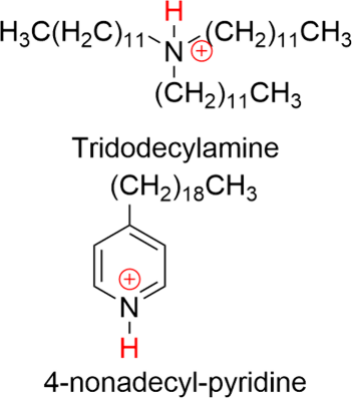
Chemical structures of two exemplary ionophores reported for local pH sensing. The position of proton exchange is indicated with a red H.

Ionophores are motivated by the working principle of the glass membrane in pH sensing. In most cases, the ionophores are (supramolecular) organic complexes which have the capability to complex protons from the solution.^51−53^ Several ionophores for ion-selective electrodes have been reported in the literature.^39^ For local pH sensing, ionophores composed of tertiary amines or heterocyclic nitrogen compounds (pyridine derivatives) have been used, where the complexation occurs via the protonation of the nitrogen center. Figure 2 illustrates one example of each of the two types of ionophores.^41,54,55^ Also other types of ionophores have been utilized for pH sensing such as hexabutyltriamodophosphate (HBTAP) for low pH,^56^ which exhibits a more complex mechanism for complexation with the protons.^36^ Since ionophores rely on ion exchange, it is necessary that they have to be highly selective to protons. Several commercially available hydrogen ionophores have proven selectivity toward protons. Newly designed ionophores need to be tested for selectivity before they can be applied reliably.^32,35,57^

In order to realize pH-sensitive electrodes, it is necessary to trap the ionophores in a polymer matrix by exploiting their hydrophobicity. Additionally, if the ionophore is neutral, some kind of lipophilic ionic additive is necessary, in order to favor the phase transfer of ions into the polymer matrix.^31^ The polymer matrix is typically made out of poly(vinyl chloride) (PVC). When working at room-temperature, plasticizers are required in order to provide good ionic mobility of the components in the polymer matrix.^31^

In addition to these classical ionophores, there is one example of a nonclassical version of ion-sensitive membrane reported for local pH sensing.^37^ Here the membrane was composed of a gel made up of the enzyme glucose oxidase in a matrix of poly(lysine), which is realized in a nanopore at the edge of a nanocapillary. The nanopore conductivity was found to vary as a function of pH, attributed to the selectivity of the membrane toward cations or anions depending on the pH. However, the conductivity was found to vary also as a function of ionic strength, limiting its general applicability. The exact mechanism of pH sensitivity has not been explained in detail. Moreover, the sensor exhibited a very low sensitivity (around 20 mV/pH) and the selectivity toward protons was not investigated in detail.

### Metal Oxides

Apart from the glass electrode, metal oxides (MOx) constitute the next frequent material class used as pH-sensitive active layer. The general requirement is that the metal oxide or hydroxide is insoluble in the pH range of interest. They can be used to detect pH by coating on a conducting electrode.^30,58^ Often, such coatings have either a mixture of oxides with two different oxidation states of the metal or an oxide with a defective oxygen stoichiometry. Several mechanisms have been proposed for pH sensing using metal oxides.^59,60^ The first one is based on ion exchange with hydroxyl groups at the surface of the oxide layer, analogous to the case of the glass electrode. Another possibility is a proton-dependent redox equilibrium between two forms of the metal oxide, each in a different oxidation state.^58,59^ The two forms may be present in a single solid phase or in two different solid phases. Apart from these two dominant mechanisms, pH sensitivity may also result from an intercalation equilibrium related to the oxygen deficiencies in the oxide layer. Finally, steady state corrosion of the oxide material can also cause a pH dependent open circuit potential. The exact mechanism is dictated by the type of metal oxide and the morphology and composition of the fabricated layers. For example, in ruthenium dioxide-based pH sensors, the dominant mechanism appears to be ion exchange at the surface hydroxyl groups.^61^ For the case of iridium oxide, the redox mechanism is more relevant.^62,63^ Typically, thick insulating layers of metal oxides are used. Therefore, potentiometry has been the main method used to detect pH response. In cases where the redox mechanism is active, the electrodes exhibit fast electron transfer kinetics.^64,65^ The simultaneous occurrence of more than one mechanism in the same metal oxide layer cannot be completely excluded. Based on these principles, the pH sensitivities of TiO_2_,^66,67^ RuO_2_,^65,68^ Al_2_O_3_,^69^ WO_3_,^70^ IrO_2_,^62,71−77^ and Sb_2_O_3_^78−81^ have been exploited in a variety of pH sensing applications. However, for local pH sensing, iridium oxide (IrOx)^71−76^ and antinomy oxide (SbOx)^78−80,82^ are the most frequently reported metal oxides as active sensing layers.

Iridium oxide can be deposited using several methods in a facile manner,^30,58,60^ e.g., by thermal decomposition of iridium chloride or reactive sputtering from an iridium target. However, for modifying UMEs, electrodeposition has been the method of choice.^71,75,76,83^ Here, a solution of iridium oxide micro/nanoparticles is prepared first by using iridium chloride as the source and oxalic acid as complexing agent at a pH of around 10.^84^ Then the sensing electrode is immersed in this solution, and iridium oxide is electrochemically deposited on the UME. A simplified form of the proton exchange with IrOx coatings involves the redox mechanism and is given by^58^
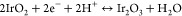
6

Thermally grown anhydrous IrOx is shown to follow the above simplified equilibrium yielding a slope close to the Nernstian limit with *m* = *n* = 2.^62,63^ Anodically prepared films are considered to be hydrous and exhibit slopes higher than 59.2 mV/pH, which is attributed to several factors including preparation conditions,^58^ hydration of the oxide coating,^85^ and pH-dependent redox mechanism,^86^ resulting in a more complex redox equilibrium. Selectivity to protons is often well-guaranteed in metal oxide coatings, provided that there are no electroactive contaminants in the oxide layer.

### Redox Probes in Monomeric Form

Molecules that undergo proton-dependent redox reactions according to equation 3 are ideal candidates as probes for measuring pH.^87^ When an electrode is covered with such molecules, the formal potential of the electrode varies as a function of pH according to equation 5. Often voltammetric methods have been used to detect the formal potential of the immobilized redox molecules. In some cases where potentiometry has been used, it is necessary that the electrode is completely covered. If this is not the case, the occurrence of a mixed potential^88^ due to the exposed areas of the underlying electrode will result in complications in the association of the measured potential exclusively to the pH value.^89,90^ Moreover, for the electrode to be selective to protons, ideally, there should be no other electroactive redox reaction occurring in the investigated electrochemical potential window.

**Figure 3:**
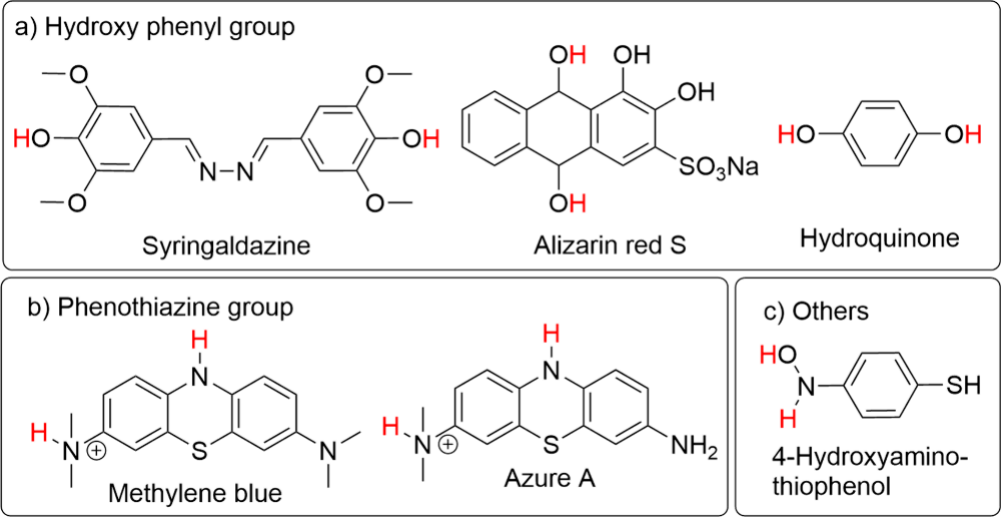
Chemical structures of selected pH-sensitive redox-active molecules belonging to the three categories: (a) hydroxyphenyl derivatives, (b) phenothiazine derivatives, and (c) others. The molecules are shown in their protonated reduced states. The position of deprotonation upon oxidation is indicated with a red H.

The probes that have been reported until now fall under hydroxyphenyl derivatives (hydroquinone, syringaldazine,^49,91^ dopamine,^92,93^ alizarin red S^94^), phenothiazine derivatives (methylene blue,^48^ Azure A), and others (e.g., hydroxy aminothiophenol, 4-HATP^50,95^). The hydroxyphenyl derivatives (Figure 3(a)) typically undergo a 2e^–^/2H^+^ redox in a broad pH range. The redox equilibrium of phenothiazine derivatives (see Figure 3(b)) is more complex, as they exhibit either a 2e^–^/1H^+^ or a 2e^–^/2H^+^ redox equilibrium depending on the pH range.^48^ 4-HATP carries an aromatic nitro group (see Figure 3(c)), which is shown to participate in a 2e^–^/2H^+^ redox reaction in a pH range of 2 to 10, when immobilized on a gold electrode.

In order that these probes can be exploited for the detection of pH, they have to be immobilized on the UME. For attachment on gold, the probes are derivatized with a thiol group (e.g., 4-HATP),^50,95^ which can be used as anchors to fix the molecule on the electrode. Simple physisorption-based methods are more common when using carbon-based electrodes, e.g., syringaldazine on carbon UMEs.^49,91^

### Redox Probes in Polymeric Form

Conducting polymers, such as polyaniline,^96−99^ polymelamine,^100^ poly(methylene blue),^101^ polydopamine,^93^ and doped polypyrrole,^102^ constitute another avenue for realizing active sensing layers. An advantage here is that such layers can be directly drafted by electropolymerization on the electrode of interest and are known to be very stable. The electropolymerization of aniline is typically carried out in an acidic solution (e.g., H_2_SO_4_) by cycling the potential several times. Polyaniline exhibits three different oxidation states and the redox equilibrium is a two-step 2e^–^/2H^+^ process.^103^ The voltammetric response of polyaniline is quite complex and hence potentiometry has been used to transduce the pH at interfaces.^98,99^

Polymelamine undergoes a clear 2e^–^/2H^+^ redox reaction in the pH range of 4 to 9.^100^ Poly(methylene blue) exhibits a complex multiple electron redox mechanism depending on the pH range,^101^ similar to the monomer methylene blue, which belongs to the class of phenothiazine derivatives. Polydopamine exhibits a similar redox behavior as dopamine, analogous to other hydroxyphenyl derivatives.^93^ However, both poly(methylene blue) and polydopamine have not yet been applied for local pH measurements.

Another method to realize a redox active layer is to incorporate the redox active molecules into a polymer matrix. For example, for pH sensing in bulk, polypyrrole (PPy) has been used, where the pH sensitivity was achieved by incorporating hydroquinone in the PPy matrix.^102^ With redox polymers, selectivity is often a critical issue, since the occurrence of several redox states in the polymer complicates the ability to unambiguously associate the sensor response to a specific redox step.

### Metals

**Figure 4:**
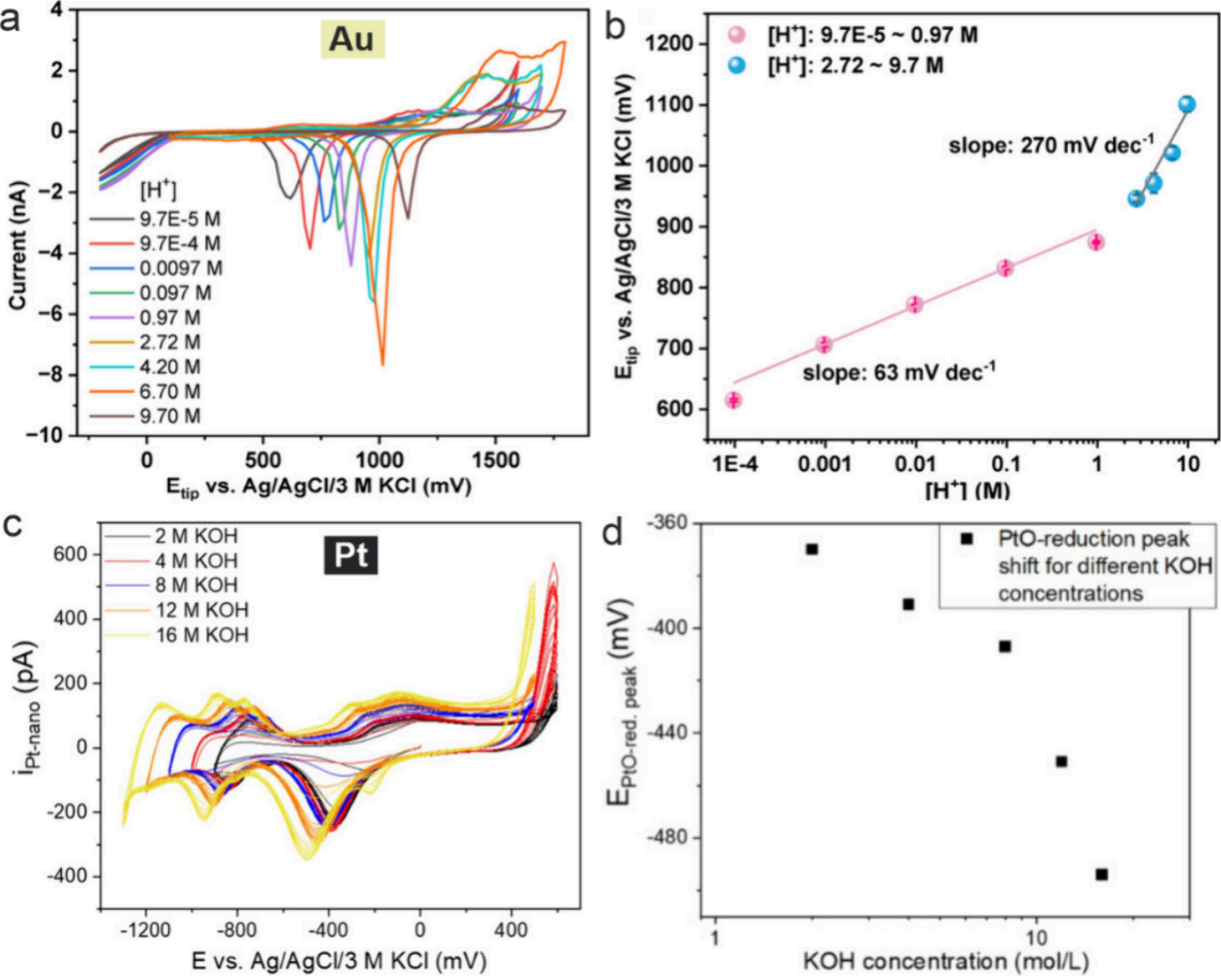
(a) CVs recorded at a gold microelectrode in aqueous solutions with varying HClO_4_ concentrations. (b) Calibration curve showing the peak position of the gold oxide reduction. Reprinted under Creative Commons License from ref (109). Copyright 2020 Wiley-VCH. (c) CVs at a Pt-UME showing the redox behavior of platinum in KOH solutions with varying concentration. (d) The reduction peaks of platinum oxide are found to shift cathodically with increasing KOH concentration. Reprinted under Creative Commons License from ref (115). Copyright 2021 Wiley-VCH.

Metal electrodes have also been proposed to directly read the local pH at selected interfaces. In general, the electrochemical oxidation of a metal, such as gold or platinum, is pH-dependent. Gold ideally undergoes a 6e^–^/6H^+^ process in acidic media and a 3e^–^/3H^+^ reaction in basic media as follows^104−107^
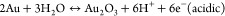
7a
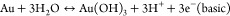
7bunder the condition that the solution is free of complexing agents. For example, in the presence of chloride, Au can be etched away by complexation to form Au(III) chloride, and hence, it is not suitable as an electrode for pH sensing. Even in noncomplexing media, the electrochemistry of gold can become complicated due to the formation of (hydrous) oxide layers.^104^ The redox processes occurring in these layers can complicate the mechanism behind the reaction equilibrium. It has been proposed that the reduction of gold oxide/hydroxide might be a one electron or two electron process in a pH range of 3 to 9.^108^ Moreover, the slope of the pH dependence of the formal potential was found to be higher than the Nernstian limit for proton concentrations higher than 1 M (see Figure 4(a-b)).^109^ Another issue is that the formal potentials for the oxidation are quite high (see typical voltammograms on gold in Figure 4(a-b)).^109,110^ Hence we are limited to buffer systems, where the components are stable at such high potentials. Nevertheless, gold UMEs and NEs have been directly demonstrated as a simple and elegant way for studying local pH in electrocatalytic reactions in perchlorate and carbonate containing aqueous solutions.^109^

Platinum also exhibits a pH-dependent formal potential for its oxidation. However, under oxidizing potentials, the Pt surface undergoes degradation,^111,112^ such as dissolution, delamination,^113^ or roughening caused by restructuring.^114^ Nevertheless, Pt UMEs and NEs^115,116^ have been used to detect local pH using a voltammetric readout. Here, the reversible formation of platinum oxide (PtOx) and its reduction are followed by voltammetry (Figure 4(c-d)). The reduction of PtOx shows a peak in the voltammogram, which depends on the pH in the vicinity of the electrode. Another alternative is the use of the diffusion-limited current of proton reduction during HER.^117^ It was claimed that this method does not poison the platinum surface and could be used to sense the proton concentration in the vicinity of electrocatalysts and biological processes. Although submicron resolution could be demonstrated, a proper calibration to estimate the pH is rather difficult since the current due to HER is intrinsically noisy due to the coevolution of hydrogen.

Apart from gold and platinum, antimony has been used as the pH-sensitive probe in one of the first local pH mapping experiments.^118^ Antimony-based UMEs have proven useful in studying local corrosion effects in metal alloys.^82,119^ Palladium, which has the capability to dissolve hydrogen is another candidate for realizing local pH sensors.^120^ After loading with hydrogen, such electrodes show Nernstian response in a broad pH range, even up to a pH of 14.^121^ Metals coated with the corresponding oxides (e.g., Ir/Ir_2_O_3_, Sb/Sb_2_O_3_) can also be exploited to realize pH sensors.^30,58^ Often such sensors have a low sensitivity since the redox processes typically involve multiple electrons. However, such sensors have not been used for local pH sensing.

Selectivity toward the desired proton-dependent redox reaction is challenging to achieve with metal electrodes. The standard potential of the redox reaction varies with the crystal orientation. Since most pH sensing metal electrodes are polycrystalline or amorphous, there will be several formal potentials at the sensing electrode for the overall redox reaction. Second, passivation of the metal surface due to oxidation may be difficult to recover. Finally, the presence of redox active adsorbates might render the electrode less selective.

## Electroanalytical Instrumentation for Local pH Sensing

For local pH sensing, it is necessary to position the pH probe as close as possible to the interface of interest. The size of the probe determines the closest distance from the interface, where the probe can be placed. It will also dictate the spatial resolution that can be achieved for the measurement of pH. On the other hand, the detection mode and the active material used will play an important role in the response time that can be achieved. Next, we look at several experimental configurations to measure the local pH. For electrocatalytic applications, also the interface of interest needs to be operated as an electrochemical cell. This brings in some constraints for how the pH probe can be deployed. Apart from using a scanning pH sensor to read the local pH, another alternative is to use rotating ring disk electrodes (RRDEs) to obtain mechanistic information about local pH in electrocatalytic reactions.

### Potentiometric Readout

**Figure 5:**
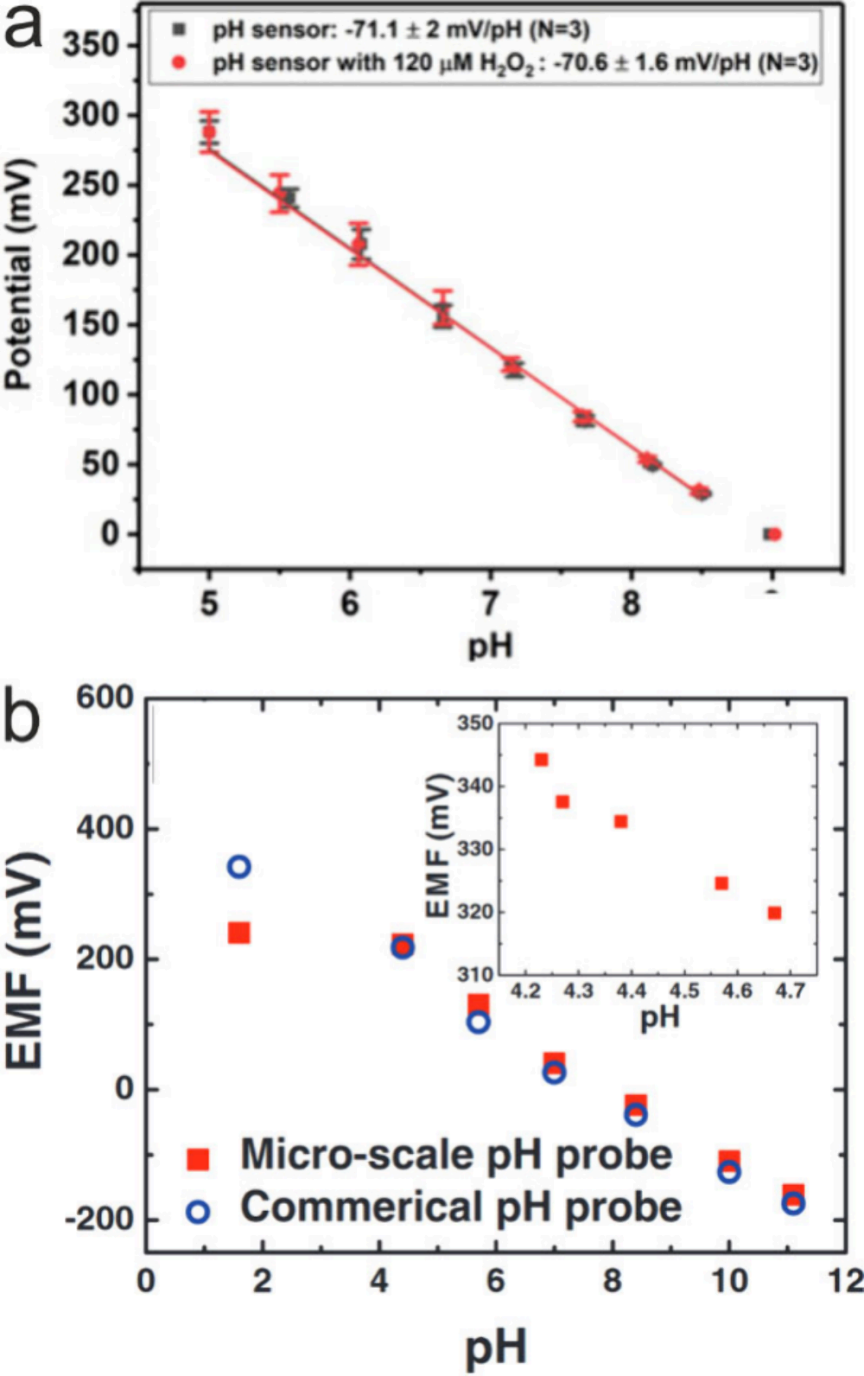
Calibration curves showing the measured open-circuit potential (OCP) as a function pH for selected potentiometric pH sensors: (a) a Pt/hydrous-IrOx (25 μm diameter) sensor with a sensitivity of 71.1 ± 2 mV/pH. The red curve shows that the sensitivity is nearly unaffected even in the presence of the interferent H_2_O_2_. Reprinted from ref (71). Copyright 2023 American Chemical Society. (b) A polymer redox sensor based on polyaniline-coated gold UME (340–600 nm diameter) showing a near-Nernstian sensitivity (56 mV/pH) in the pH range of 4–12. EMF: Electromotive Force = OCP. Reprinted with permission from ref (98) Copyright 2013 Electrochemical Society.

In both detection principles outlined earlier, the potential of the sensing electrode varies as a function of pH. Hence, by measuring the electrode potential of the pH probe against a stable reference electrode, we can estimate the pH after appropriate calibration. A two-electrode configuration is sufficient for this readout. The configuration with the membrane potential is best suited for applying potentiometry since the membrane appears clearly in series in the circuit. The pH sensitivity arises due to proton exchange at the membrane-solution interface, which is reported to be intrinsically very fast.^122^ As the membrane potential appears in series, high membrane impedance due to the small electrode geometry or inhomogeneities in the membrane may limit the response speed in this readout scheme.^122^ When redox-active monomers or polymers are used, it is necessary that the molecules are firmly attached on the electrode surface and that the electrode is completely covered to avoid the occurrence of a mixed potential. Figure 5 shows some examples of potentiometric calibration curves using sensors based on MOx and a redox polymer. Often the obtained sensitivity is very close to the Nernstian limit. The best response times are in the subsecond range.^41^

### Voltammetric Readout

**Figure 6:**
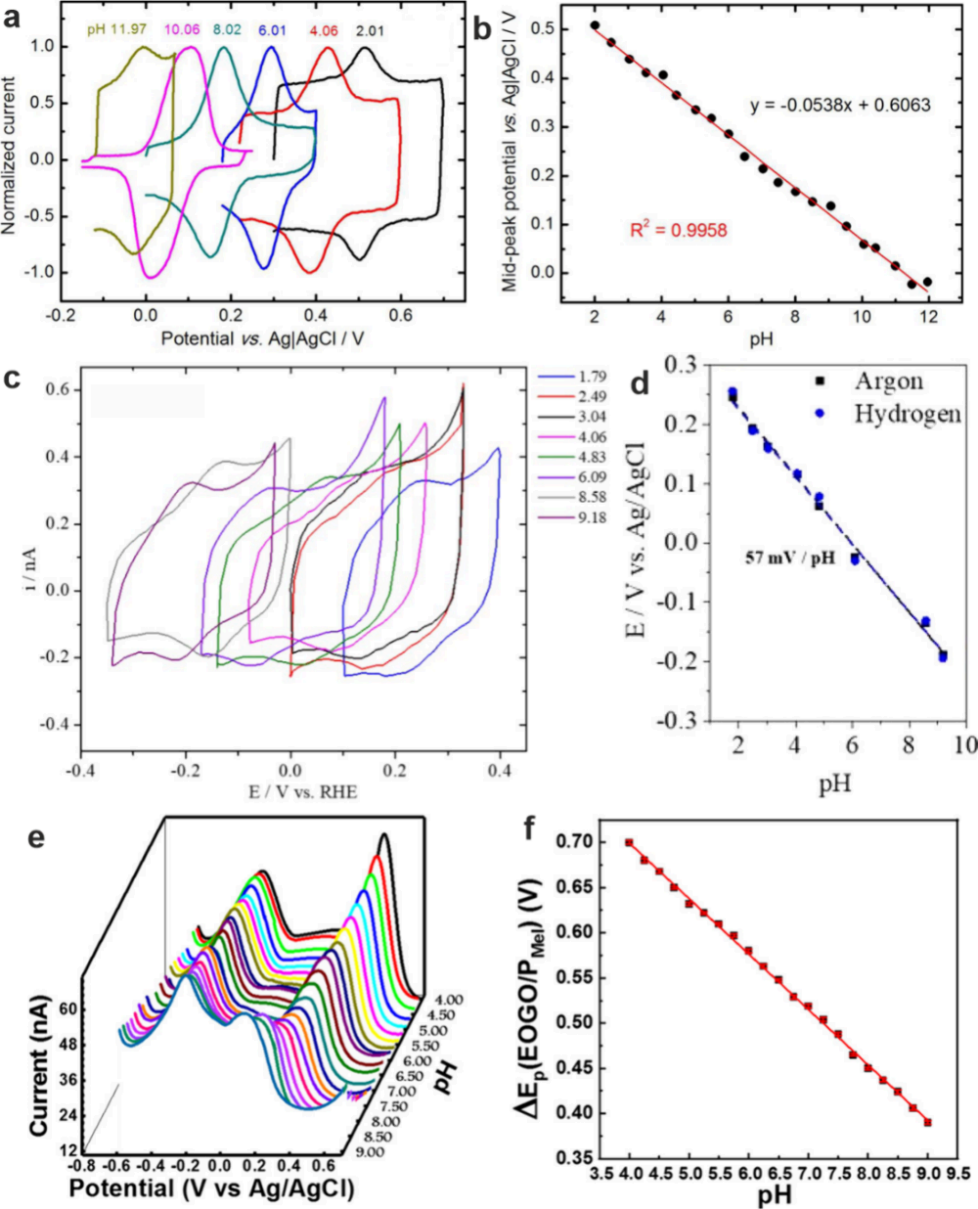
(a) CVs recorded at a syringaldazine-modified carbon nanoelectrode (50 nm diameter, pH range: 2.01–11.97). (b) Calibration curve showing the midpeak potential as a function of pH. Reprinted from ref (49). Copyright 2015 American Chemical Society. (c) CVs measured at a 4-HATP-modified Au-UME (50 μm diameter, pH range: 1.79–9.18). (d) Calibration curve of the corresponding peak potentials obtained in solutions saturated with Ar or H_2_. As the aim was to study hydrogen evolution, the tip was also tested in H_2_-saturated solution to demonstrate the stability of the sensor against H_2_. Reprinted from ref (50). Copyright 2020 American Chemical Society. (e) DPVs measured at a carbon fiber microelectrode modified with graphene oxide and polymelamine. Data obtained in buffer solutions of varying pH from 4.0 to 9.0. The peak at around −0.22 V corresponds to the pH-independent redox of graphene oxide, while the second peak in positive potentials is due to the pH-dependent redox of polymelamine. (f) Calibration curve showing the peak spacing as a function of solution pH. Reprinted from ref (100). Copyright 2022 American Chemical Society.

In the configuration using voltammetry (VA), the current is measured as a function of potential in either a two- or three-electrode configuration. For local pH sensing using UMEs and NEs, the current levels are in the sub-nA range. Here, a two-electrode readout is sufficient since the low current level is hardly expected to affect the electrode potential of the reference electrode. For local pH sensing, cyclic voltammetry (CV), linear sweep voltammetry (LSV), or differential pulse voltammetry (DPV) has been utilized. Examples of response curves based on CV using monomeric and based on DPV using polymeric redox species-based UMEs are shown in Figure 6. In contrast to the potentiometric readout (where the potential is directly proportional to the pH value), the VA curves need to be further analyzed in order to extract the formal potential. There are some challenges in this analysis procedure. In CVs and LSVs, it is important to devise methods to overcome problems due to electrode capacitance or ohmic drop. These aspects render peak potential estimation difficult. Such problems are rarely discussed in detail in reported works. The instrumental readout time is governed by the scan rate used.

### Electrified Interfaces

One of the application avenues for local pH sensing is the in situ study of electrochemical processes at a range of surfaces during operation. In this case, the surface of the electrode of interest is polarized under electrochemical control and is the working electrode in a three-electrode electrochemical cell, either as part of a potentiostatic, galvanostatic, or potentiodynamic measurement. At such electrified interfaces, special care has to be taken in the design and use of the pH probe.^79,123−125^

The probe has to be very selective to protons. Since the pH probe in a two-electrode configuration is also an electrochemical cell, it needs to be ensured that the extracted pH information (through potentiometry or voltammetry) is not masked by the reactions occurring at the electrified interface of interest. To avoid such a misinterpretation, a bipotentiostat can be used, where two working electrodes (one for the pH probe and one for the electrified interface) can be operated with the same counter and reference electrodes placed in the electrolyte solution.^71^ Another common strategy that has shown success is to just consider the two cells as isolated and use two different potentiostats—one for the EC-cell with the surface of interest and the other for the EC-cell with the pH probe.^50,83,126^ For measuring the current or potential at the UME pH probe, an electrometer or voltmeter can be used in place of a potentiostat due to the two-electrode nature of the cell.^83^

An important issue when measuring local analyte concentration using potentiometry is the occurrence of a spatially varying electric field induced by the polarization of the electrified sample interface.^23,127^ This effect causes a modulation in the local potential depending on where the sensing electrodes are placed.^127,128^ One way to minimize this effect is to construct the two-electrode pH probe with the sensing and the reference electrodes placed very close to each other, e.g. in a double-barrel or triple-barrel pipet.^80,128^ Another strategy is to incorporate the reference electrode for the pH probe inside the capillary, in configurations where this is possible,^98,123^ e.g. with ionophore membranes. Motivated by design strategies for electrochemical detectors in capillary electrophoresis,^129^ it has been proposed that the working and counter/reference electrodes of the pH probe may be placed on an equipotential surface.^79^ This is expected to maintain the integrity of the pH probe sensor signal and be less affected by the field distribution induced by the electrochemical cell at the electrified interface. For accurate pH sensing, it has to be ensured that the electric field effects are mitigated by placement of the reference electrode close to the pH sensing electrode.^23,127^

It is also important to ensure that the pH probe only minimally affects the diffusion profile of the electrode reaction occurring at the surface of interest.^125,130^ Moreover the pH probe must be accessible to protons, ideally without any mass transport limitation. The extension of the diffusion profile is typically of the order of the diameter of disk UMEs.^42^ Hence the operating height of the pH probe above an electrified interface is dictated by the size of the pH probe. Using sharp tapered NEs is more advantageous. For such electrodes, the extent of the diffusion layer is in the submicron range and can be placed very close to the investigated surface.^76,98^ This also helps to attain a good spatial resolution in the submicron range. The hindrance in diffusion due to the presence of the pH probe will cause unreliable signals, which could result in a wrong estimation of local pH.^125^ One way to correct for this error is the use of simulations using finite element or finite difference methods.^130^ Alternatively several control experiments may be carried out to verify that diffusion hindrance is minimal at the desired operating height.

### Fabrication Strategies for Micro- and Nanoelectrodes

**Figure 7:**
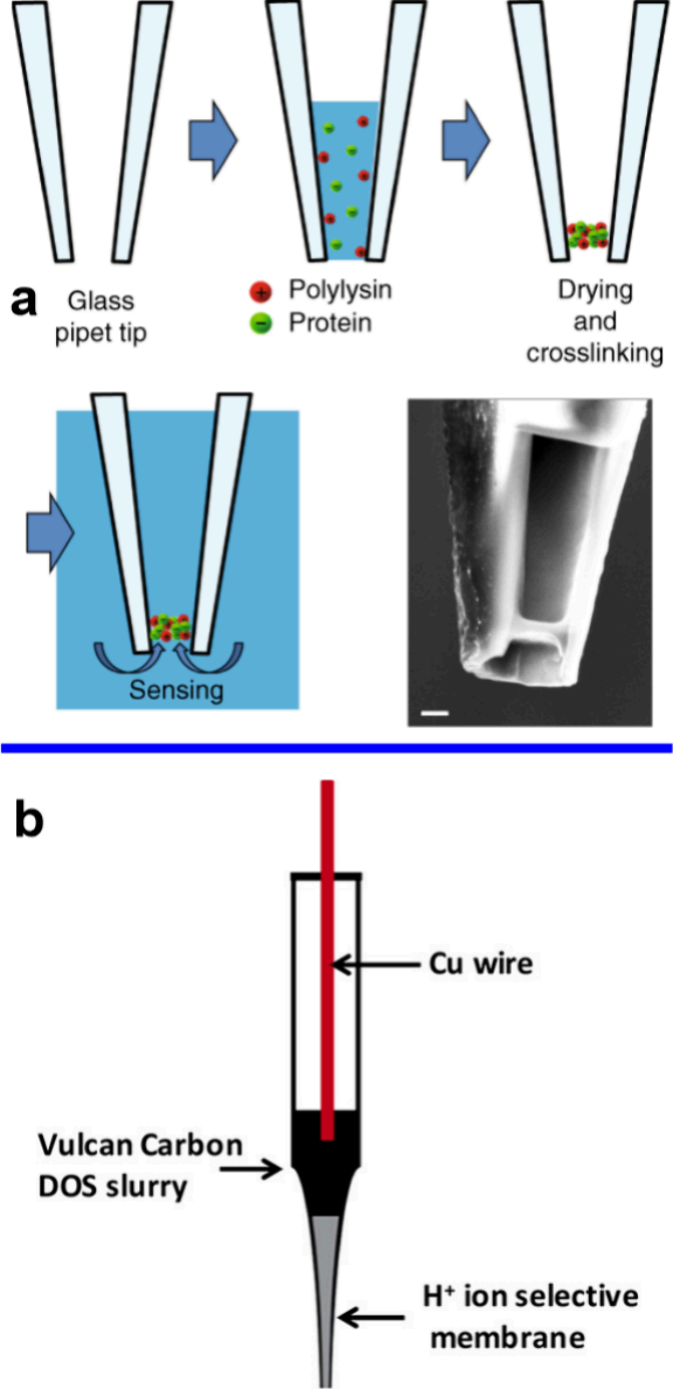
Ionophore-based single-pH-probes. (a) Schematic showing the fabrication of a membrane in a nanopore at the end of a nanocapillary. The membrane comprises the enzyme glucose oxidase and poly(lysine). (right bottom) SEM image of the end of the nanocapillary showing the dried membrane close to the end of the capillary. Scale bar 500 nm. Reprinted under Creative Commons License from ref (37). Nature Publishing Group. (b) Schematic of a pH microprobe using a solid contact between a carbon paste and the proton selective membrane. DOS: dioctyl sebacate. Reprinted from ref (41). Copyright 2017 American Chemical Society.

Local pH sensing experiments require UMEs or NEs that can probe the interface of interest with high spatial resolution. Such electrodes may be composed of a standalone single sensing electrode or multiple sensing electrodes bundled together. In order to realize ionophore-based pH UMEs or NEs, the tip of a pulled capillary (glass or quartz) is back-filled with the ionophore cocktail to form the proton-selective membrane (see Figure 7(a)).^37,41^ For realizing pulled capillaries, a laser pipet puller is typically used. Here the pulling parameters need to be optimized in order to reproducibly obtain capillaries with tips of a desired diameter.^131^ After the polymeric membrane with ionophores is formed, the capillary is back-filled with the internal solution and a reference electrode inserted in order to realize the pH sensing electrode.^53,55,132^ Alternatively, a solid contact can be established to the membrane using a conducting material such as carbon paste (see Figure 7(b)) or carbon fibers.^41,52,80^

**Figure 8:**
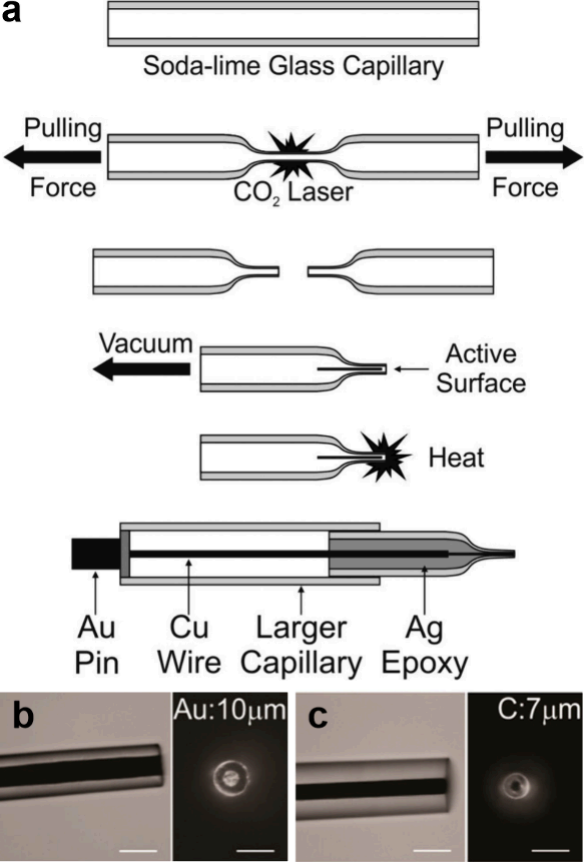
(a) Schematic showing the fabrication protocol for metal UMEs: Sodalime glass capillaries are heated, while a pulling force is applied to the ends using a P-2000 micropipette laser puller, resulting in tapered micropipette tips. A metal wire is inserted into the pulled micropipette tip and sealed using a heating a coil. Finally, the micropipette tip is assembled with external electrical connections. Subsequently, the tip is polished to expose the embedded metal wire (not shown). (b, c) Side view and end view of typical pulled capillaries resulting in disk UMEs—Au wire of 10 μm diameter (b) and carbon nanofiber with diameter of 7 μm (c). Scale bar 25 μm. Reprinted from ref (135). Copyright 2015 American Chemical Society.

For realizing other pH sensing probes, metal- or carbon-based UMEs or NEs are first fabricated. Subsequently they are modified with redox active monomers^91,133^ or polymers^98^ or coated with metal oxide films.^134^ The main requirement for realizing the UME or NE is that only the end of the electrode is exposed to the solution while the rest in insulated. A simple technique to achieve this is to insert a metal wire or a carbon microfiber inside of a pulled glass capillary and seal it by heat (see Figure 8(a)).^50,130^ The edge of the wire is then exposed by a polishing step using grit paper. Using this technique, it is possible to obtain a disc-shaped electrode with diameter in the range of the wire diameter typically down to about 10 μm (see Figure 8(b,c)).

Another common strategy is to use the laser puller after inserting the metal wire into a normal capillary.^131,135−137^ The glass capillary (soda lime glass, borosilicate, quartz) with the metal wire is first thinned by simultaneously applying a small pulling force while heating the capillary using a CO_2_ laser.^135,136^ The prerequisite is that the applied temperature is close enough to the melting point of the metal but small enough to keep the capillary stable. Usually the electrode is connected to a copper wire as an electrical contact using silver-filled epoxy glue before inserting it in the glass capillary. Prior to heating, the capillary is evacuated from both sides using a vacuum pump. The resulting electrode diameter can be in the range of a few microns down to around 10 nm.^136^

The pulling procedure inevitably results in a metal tip sealed with glass passivation due to heat. The tip must, therefore, be polished to achieve a well-defined electroactive surface area. As with the previous method, a mechanical polishing procedure using a polishing plate/grit paper and an alumina powder is carried out.^136^ An alternative method utilizes dipping in HF to etch the sealing glass in order to expose the nanoelectrode. However, this method yields tips with conical geometries.^131^ Due to the lower melting point of gold (1064 °C) in comparison to Pt (1768 °C), the fabrication of Au UMEs needs to be adapted a bit. They need to be prethinned before pulling to avoid discontinuous beading of the wire.^137^

**Figure 9:**
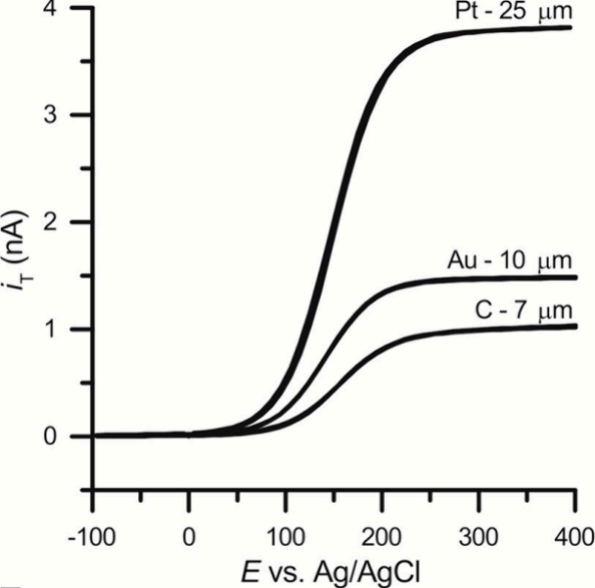
CVs measured at the metal and carbon disk UMEs described in Figure 8. The diameters of the electrodes are as indicated. The CVs were obtained in 1 mM ferrocenemethanol in 0.1 M KCl at 10 mV/s. Reprinted from ref (135). Copyright 2015 American Chemical Society.

**Figure 10:**
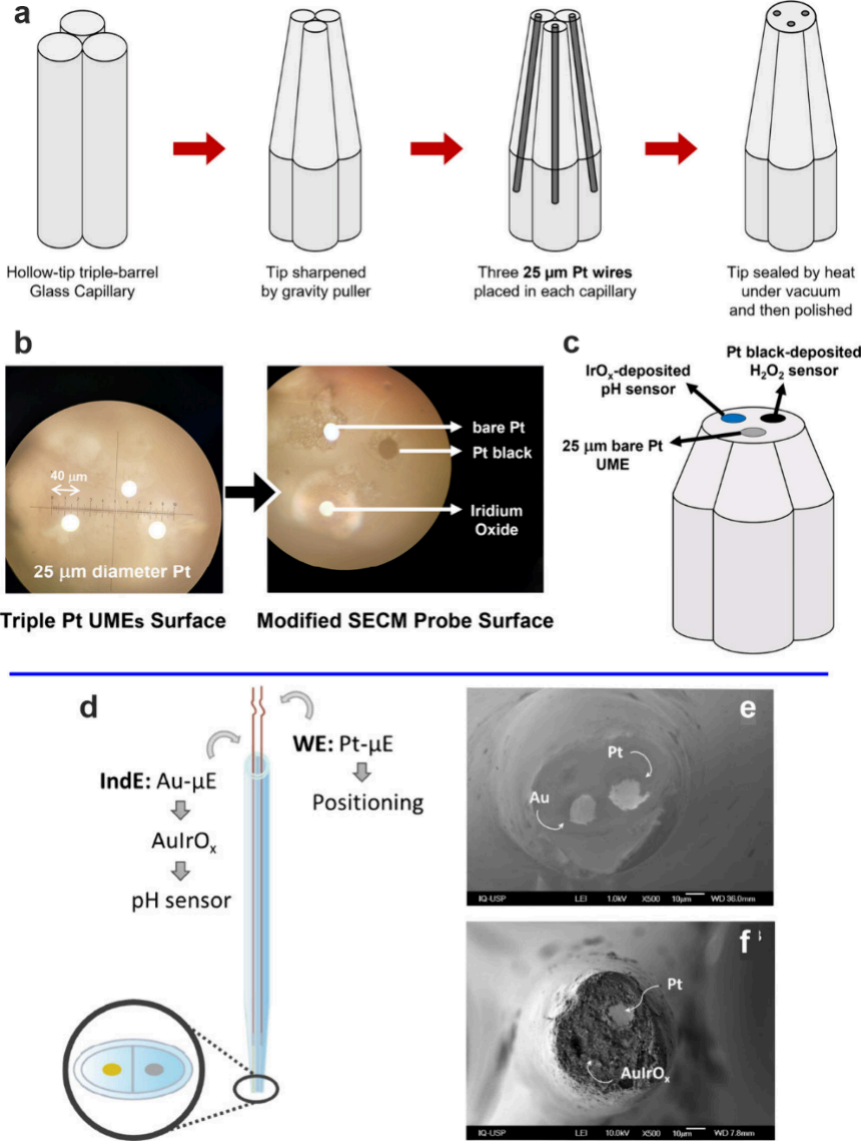
(a-c) Triple Pt-based pH-H_2_O_2_ probe. (a) Schematic showing the fabrication of the triple probe tip. (b) Optical images of the surface of the tip before and after modification with Pt black (for H_2_O_2_ sensing) and IrOx (for pH sensing). (c) Scheme of the fabricated triple probe tip. Reprinted from ref (71). Copyright 2023 American Chemical Society. (d–f) Dual Au/Pt-based pH probe. (d) Scheme of the dual probe, with the working electrode (WE) used for positioning the probe above the surface and the indicator electrode (IndE) for pH measurement. (e, f) SEM images of the Au/Pt-dual UME before (e) and after (f) electrodeposition of IrOx. Reprinted with permission from ref (74). Copyright 2023 Elsevier.

The characterization of the fabricated electrode is carried out using cyclic or linear sweep voltammetry with an outer sphere redox probe (see Figure 9). The diameter of the disk electrode can be estimated from the steady state current (*i*_SS_), given by^135,138^*i*_*SS*_ = 4π*nFDaCβ*, where *n* is number of electrons transferred, *D* the diffusion coefficient, *F* the Faraday constant, *a* the radius of the disk, and *C* the bulk concentration of the redox active species. β a tabulated factor^138^ determined by the geometry of pipet tip. Multiple electrodes on the same sensing probe can be obtained by starting out with double barrel or triple barrel capillaries and using the laser pulling method with or without metal wires (see Figure 10).^71,74,76,134^ Alternatively, two separate capillaries are joined together before performing the laser pulling.^80,128^

**Figure 11:**
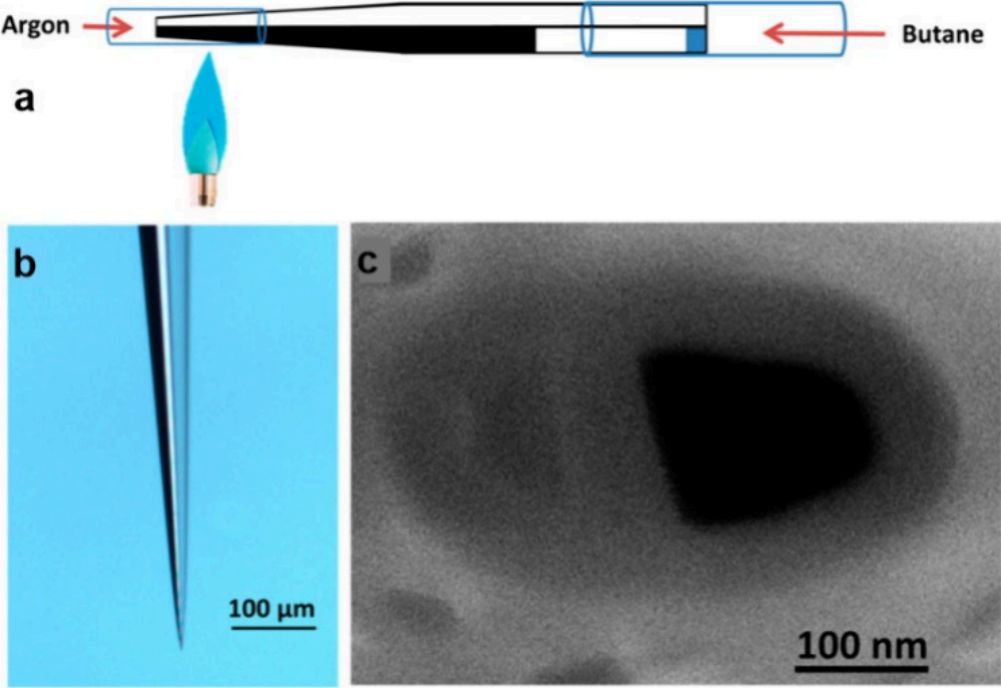
(a–c) Dual SICM-pH-probe. Schematic showing the principle of carbon deposition in one of the capillaries of a pulled theta/double barrel pipet. (b) Optical and (c) SEM images of a double-barrel pipet after carbon deposition. The carbon electrode is modified with IrOx to realize the pH probe. The unfilled capillary is used for SICM-based height measurement. Reprinted from ref (76). Copyright 2013 American Chemical Society.

Carbon-based UMEs and NEs can also be fabricated using this procedure by replacing the metal wire with a carbon fiber. Achievable diameters are in the range of a few microns.^91^ An alternative strategy utilizes the pyrolysis of a carbon source to deposit carbon from the gas phase inside a pulled capillary (see Figure 11).^49^ Until now there are only a couple of examples where carbon-based electrodes were used for local pH sensing.^49,91^

### Scanning Probe Microscopy

In order to position the pH probe close to the surface of interest, a scanning (electrochemical) probe microscope (S(E)PM) can be deployed.^139^ Several variants of S(E)PM have been demonstrated, among which the Scanning Electrochemical Microscope (SECM), the Scanning Ion Conductance Microscope (SICM), and the scanning ion-selective electrode technique (SIET) are quite common.^23,51,118,132,140,141^ The advantage with S(E)PM is that in addition to local pH information, topographic information and, in some cases, chemical information can also be gathered, which can all be correlated with each other.

Generally, in such setups, a scanning probe with the sensing electrode (often called the tip) is scanned over the surface of interest, and a desired surface-related signal is measured as a function of the spatial coordinates *X* and *Y*. The tip–surface distance is maintained constant through a feedback signal. For example, in SICM, the ion current through a nanopipette tip is maintained at a certain level in order to keep a constant distance of the tip to the surface.^139,142^ In the simplest form of an SECM, a redox probe needs to be present in solution. The redox cycled current between the probe tip and the surface can be used as a feedback signal to maintain the height.^140^

The feedback signal does not have to be an electrochemical signal. A more elegant way to control the probe positioning on the surface is to use shear force control.^53,55,109,133,143^ Here lateral oscillations are induced in the pH sensor tip coupled to a shear piezo. As the tip approaches the surface, the amplitude of oscillation is damped, which can be detected by using a second piezo element. A set signal amplitude is used as feedback to control the height of the tip above the surface. This approach has the advantage that there is no crosstalk between the feedback signal and the signal recorded for extracting the local pH.

By contrast, in the case of SICM, SIET, and current-feedback-controlled SECM, at least two sensors are necessary on the tip, one for controlling the tip–sample distance and the other for pH sensing.^51^ Hence, in general at least two probes are integrated on the scanning tip.^71,72,76,78,80^ For example, it was possible to measure local pH together with topographic information in a corroding calcite crystal.^76^ This was achieved by using a double barrel nanocapillary, with one barrel used as a SICM probe and the ionic current used as the feedback signal. The other barrel was filled with pyrolytic carbon followed by deposition of IrOx, which functioned as a pH probe (see Figure 11) Using a dedicated feedback signal allows, in general, the positioning of the nanoelectrode tip as close as 100 nm to the sample surface. In this manner, changes in the topography could be clearly correlated with the dissolution rate of the calcite crystal. Theoretical modeling has been exploited to understand the pH distribution in the vicinity of the sample surface.

The multiprobe tip may also be realized with SECM electrodes.^71,74,78,80,99^ In this case, one of the probes is used to control the height of the tip above the surface. Additional probes on the tip allow the characterization of ancillary chemical species e.g. hydrogen peroxide^71^ or reactive nitrogen species^72^ or other ions.^80,144^ This can help provide a deeper insight into reaction mechanisms at the solid–liquid interface. Figure 10 presents two examples of such multiprobe tips. Bulk micromachining has also been used to realize double probes^72^ on the scanning tip. This however has a poorer spatial resolution than the UME probes. While multiprobe tips bring a high degree of versatility to the measurement setup, it is not always straightforward to realize such electrodes at the nanoscale reproducibly. Often, they require detailed characterization on a probe-by-probe basis in order to enable an unambiguous interpretation of the measured currents.

Where dual probes were not available, the pH UME has itself been used in a SECM configuration to position the tip as close as possible to the sample surface.^91,109^ Here, it is necessary to first estimate the position of the surface using a scan in the vertical direction in the presence of a redox probe. After the probe is placed at a desired distance, the solution needs to be exchanged in order to remove the redox probe. Subsequently the pH measurement is carried out.^41,145^ Compared to the multiprobe strategy, this is a rather tedious method. Moreover, it requires the redox probe and replacement of solution, during which high stability must be ensured such that there is little change in the tip–sample distance.

Another elegant way to avoid the use of multiple probes is to use an AC signal as feedback to position the probe tip above the surface to be investigated. In this case, the capacitance derived from the AC signal has been successfully used to position the tip at a desired height above the sensor surface.^50,126^ The AC signal is applied in addition to the potential signal applied to the voltammetric pH probe. This methodology has the advantage that a redox active species is not present in the solution. Another possibility is to perform the tip positioning and the pH sensing in two separate steps, with some kind of alignment between the two different steps.^79,130^ For example, profiling wells were placed on top of the sample to repeatedly position the different sensing electrodes at the same location.^79^ Alternatively, the tip–sample distance is first mapped by using a capacitive signal with the pH electrode in air. In a second step, the tip is placed at a calibrated height in the solution before the pH measurement is performed.^130^ With this strategy, the achievable positioning accuracy is on the order of the electrode diameter.

### Rotating Ring Disk Electrodes

**Figure 12:**
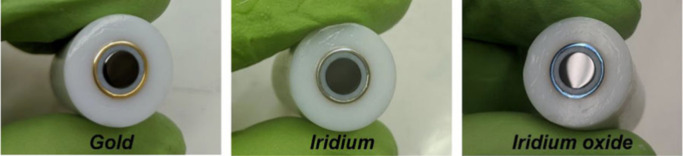
RRDE: Photographs showing an RRDE with gold disk and ring (left), after Ir metal deposition on the ring (middle), and after conversion to IrOx (right). Reprinted from ref (73) .Copyright 2022 American Chemical Society.

The use of a RRDE configuration is a suitable strategy to overcome some of the problems outlined above for the local detection of pH at electrified interfaces.^4,146^ RRDEs are widely deployed for studying electrochemical kinetics, where mass transport limitations are strongly minimized. The general idea of using RRDE to detect chemical species was proposed already in the 1980s.^146^ An electrochemical reaction occurring at the rotating disk electrode induces a product flux toward the probing ring electrode. For local pH sensing, the disk comprises the material or contains the system that needs to be investigated, while the pH sensor is realized as the ring electrode (see Figure 12).^73,75,147^ Having the pH sensor away from the electrified interface ensures that the pH measurement does not affect the reactions taking place at the surface of interest.^95^ Moreover, there is no mass transport limitation due to the presence of the pH sensor.

In this context, metals or metal oxides are ideally suited as ring electrodes for pH sensing.^4,73,75,146,148,149^ Also, redox active monomers/polymers have been utilized as pH sensing ring electrodes.^95,150^ The readout in RRDE systems is typically handled by a bipotentiostat, which can be directly used to record the ring current or potential and estimate the pH through appropriate calibration. One disadvantage with RRDE is that we cannot obtain spatial electrochemical activity profile using this methodology. However, the hydrodynamics and the distribution of chemical species can be modeled well,^151^ which has enabled the accurate estimation of pH at the disk surface based on pH measurements at the ring.^73^ If the main goal is just to obtain mechanistic information about an electrochemical reaction using a homogeneous material at the disk, this configuration is ideally suited.

## Proof-of-Concept Applications

We focus here on three application areas, where the use of local pH sensing appears promising to gain additional or complementary information regarding the mechanistic details of interfacial reactions or processes: electrocatalysis, corrosion, and biointerfaces. In addition, the local pH obtained in a spatially resolved manner can be correlated with corresponding morphological or chemical information on the surface being investigated. The information gained can help engineer the interface for an application of interest.

### Electrocatalysis

Many reactions relevant in electrocatalysis such as the hydrogen evolution reaction (HER), oxygen reduction reaction (ORR), and CO_2_ reduction reaction (CO2RR) are coupled with protons.^152−156^ Information about the interfacial pH is expected to strengthen our mechanistic understanding of these reactions.^157^ Since in many cases the electrocatalyst surfaces are rather heterogeneous, it is vital to relate the operation and efficiency of electrocatalysis to surface morphology or composition. Hence it is important to obtain spatially resolved information about chemical species formed or consumed during an electrocatalytic reaction. Thus, local pH mapping in operando will help in understanding structure–property relationships at nanostructured electrocatalyst surfaces. Such an understanding can help improve their design and thereby optimize their efficiency and performance.

**Figure 13:**
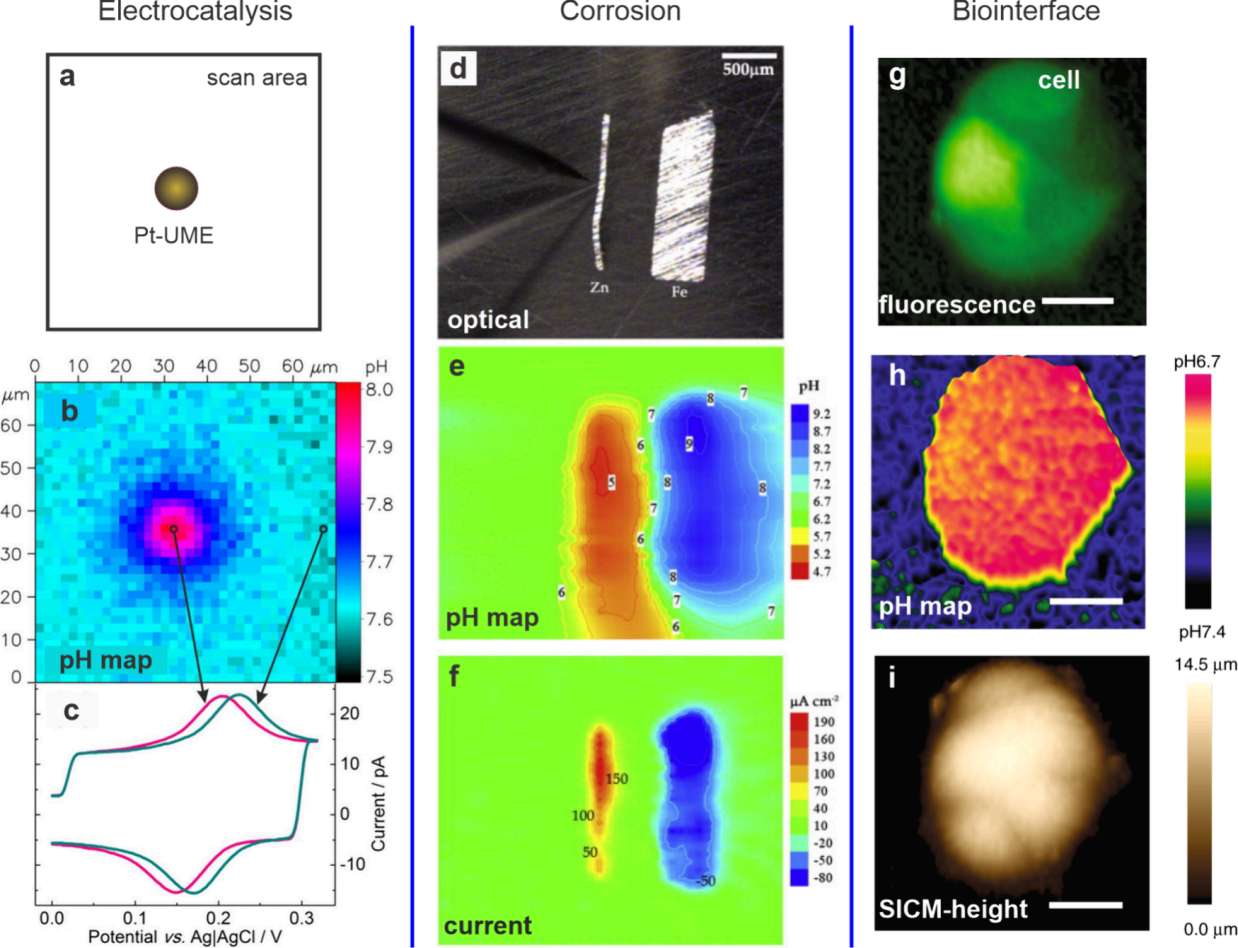
(a–c) Local pH map at a Pt-UME measured using a syringaldazine-modified carbon nanoelectrode. (a) Layout of the measurement showing the imaging area containing a Pt-disk in the middle. (b) A map of pH recorded 2.5 μm above the 10 μm diameter Pt disk electrode. The electrode is held at a potential of −0.8 V promoting oxygen reduction. (c) Example CVs at the pH electrode at a scan rate of 0.66 V/s, from which the pH is extracted. Reprinted from ref (49). Copyright 2015 American Chemical Society. (d–f) Local pH map at a corroding Zn–Fe couple. (d) Optical image, (e) pH map recorded using a pH membrane UME, and (f) and current density recorded using a vibrating Pt–Ir probe. Reprinted with permission from ref (51). Copyright 2011 Elsevier. (g–i) Mapping of pH_e_. (g) Fluorescence image, (h) pH map, and (i) SICM-height image of a low-buffered MCF7 breast cancer cell in an estradiol-deprived medium. The pH and SICM-height were obtained using the dual SICM-pH probe shown in Figure 11. Scale bar 20 μm. Reprinted under Creative Commons License from ref (37). 2019 Nature Publishing Group.

There are several examples where local pH changes have been clearly observed at the vicinity of a polarized Pt electrode in various forms—pH increase in the case of ORR (see Figure 13(a-c))^49,158^ and HER.^50^ These results serve as a proof-of-principle demonstration of the successful use of local pH probes to detect the progress of electrocatalytic reactions. Moreover, using local pH measurements, new mechanistic information could be obtained in CO2RR. The oxidation of CO generated by electroreduction of CO_2_ at a gold electrode was monitored in SECM format.^126^ The occurrence of two peaks in voltammetric CO electrooxidation could be attributed to differences in local pH. The local pH was, however, measured separately from the local monitoring of the CO oxidation reaction. In addition to mechanistic details, information about the dynamics of different chemical species (CO and CO_2_) in the diffusion layer could be followed by an appropriate choice of applied potential profiles.^130^ Local pH sensing has also been used to gain insight in to the mechanism of hydrogen egress from a hydrogenated Pd surface at resolution of around 30 μm.^52^

Using RRDEs to study electrocatalysts is still in its infancy. While measuring electrocatalysts for ORR, HER, and CO2RR, it could be shown that at varying current densities, rotation speeds, or RRDE geometries, the changes in local pH largely followed expectations based on known mechanistic details.^73,75,95,148,150^ These proof-of-principle experiments may be extended to other electrocatalyst systems and are expected to provide new insight into local pH variations constituting a mechanistic step in electrocatalytic reactions.

### Corrosion

Corrosion of bulk metals is an important issue in several applications where the metal surface is exposed to humidity or an aqueous environment with high salt.^11^ In nanostructured surfaces, nanoparticle dissolution or degradation is an important issue, which must be suppressed for an optimal application. In both cases, metals going into solution as metal ions (which act as weak Lewis acids) tend to acidify the local environment.

Corrosion on surfaces is often heterogeneous in nature, which is sometimes attributed to the roughness of the metal surface.^11,159^ Local sensing of chemical species is suited for studying corrosion mechanisms on heterogeneous metal alloys.^8^ Spatial differences in corrosion rates in alkaline media could be associated with local pH variations and can be correlated with local surface heterogeneities or variations in chemical composition or dynamic occurrence of reactive sites on the alloy surface.^78,80^

This knowledge can help in the design of appropriate passivation techniques to minimize corrosion. Moreover, pH mapping can be used to screen the efficacy of surface passivation layers.^53^ For example, using a shear force SECM, the role of local pH during the precipitation of a sealing overlayer on anodized aluminum oxide could be assessed. Thus, quantitative information could be gathered regarding the surface treatment process for corrosion protection. Local pH measurements provide evidence that corrosion at localized areas (e.g., in zinc-coated steel) increases with immersion time in aqueous environment.^141^ Moreover, the passivation film (e.g., iron hydroxide film or PEDOT: poly(3,4-ethylenedioxythiophene) is found to breakdown in localized areas due to corrosion induced e.g. by the presence of chloride ions.^160,161^

Local pH detection during corrosion has been reported on magnesium or aluminum based alloys and stainless steel, which are commonly used in several engineered systems e.g. as battery electrodes, in aircrafts or in biodegradable implants.^53,80,132,160,162^ Magnesium is used often as a sacrificial anode due to its extreme standard potential. Understanding corrosion at a microscopic level is fundamental to engineering corrosion resistance in the designed parts. S(E)PM-based instrumentation outlined earlier has been used to obtain local chemical information on model surfaces during corrosion (see Figure 13(d-f)).^51,76,162^ When such analytical methodologies are extended to realistic systems, it can be expected that more information regarding the mechanism of corrosion and corrosion inhibition can be gathered.

### Biological Interfaces

Local pH sensing is also beneficial to the study of biological interfaces. The simplest application is to study changes in local pH at the vicinity of a cell or cell membrane.^98,118^ In this context, local pH sensing can be used as a screening platform to study the metabolism of cells when they are subjected to different kinds of chemical or biological triggers. Since the changes are expected to be mainly at the vicinity of the cell surface, some method of feedback-controlled positioning is necessary. Using a dual SECM probe, a pH decrease could be observed at the vicinity of myoblast cells when exposed to caffeine.^74^

Microscale pH sensing has been exploited in understanding the local activity of biofilms composed of different bacterial species such as *Leptothrix discophora* SP-6, *Streptococcus gordonii*, and *Streptococcus mutans*.^41,163^ However, it was necessary to detect also other chemical species (e.g., H_2_O_2_ or other redox active species)^71,123^ in order to correlate local variations in pH with bioactivity and metabolite-driven cell behavior.^164^ Microscale pH measurements have also been applied on cathodic biofilms in order to understand the role played by pH on the redox activity of microbial species.^123,163^

Another important parameter is extracellular pH (pH_e_), which controls several cellular processes, especially in cancer cells. pH_e_ is found to be lower in the vicinity of cancer cells, which promotes cancer cell proliferation.^7,91,165^ Using a syringaldazine modified carbon microelectrode, indeed a gradient in pH could be deciphered at the vicinity of cancer cells.^91^ With a dual SICM-nanopore tip, pH_e_ could be mapped on the surface of cancer cells (see Figure 13(g-i)).^37^ This information is crucial for the development of protocols for drug delivery and cancer cell imaging. The intrinsic heterogeneity of cells and cell-to-cell variation are challenges to be overcome for designing appropriate therapeutic approaches.^166^ Here, local pH sensing may prove to be helpful in handling this complexity.

## Challenges

Most of the reports on local pH sensing are proof-of-principle demonstrations of the possibility to use a miniatured pH sensor to measure proton activity at a solid–liquid or bio interface. For exploiting the full potential of this analytical approach, several challenges must be overcome.

### Sensor Performance

As for every sensor, the key parameters for local pH sensors are sensitivity, selectivity, repeatability, and stability. For potentiometric sensing, most sensors already have shown the best sensitivity achievable, i.e., very close to the Nernstian limit. There are some IrOx sensors claiming super-Nernstian sensitivity.^71,74−76,145^ In such cases, the underlying mechanism of the redox reaction is not yet unambiguously clarified, which could be, for example, due to the occurrence of the hydrous oxide or mixed oxide species. Here, the *m* and *n* values in equations 3 to 5 are just assumed to be equal. In order to properly classify an observed high slope value (greater than 59.2 mV/pH) as super-Nernstian, it is necessary that we have a clear idea of the reaction mechanism.

In the case of voltammetric detection, the goal is also to estimate the formal potential of the redox reaction. Also here, the best sensitivity we can achieve is the Nernstian limit. However, for estimating the formal potential, the Faradaic current due to the redox reaction must be stronger than the noise level and distinct from the non-Faradaic capacitive background. This will have an effect on the pH resolution that can be achieved. UMEs and NEs are expected to show minimal non-Faradaic currents.^44^ However, the reported CVs often contain a significant capacitive contribution (Figure 6). Minimizing this contribution through appropriate electrode fabrication techniques will be required to improve the pH resolution.

Selectivity of the sensor is an important aspect that has been neglected in several studies. As is usually the case, the selectivity is mainly dictated by the active material, which was discussed above separately for every material/molecule class. Especially, when using local pH probing at electrified interfaces, it has to be ensured that the current and potential responses are due to proton activity and not due to other chemical species. Stability and repeatability are other important parameters. Potentiometric pH sensors are very much prone to drift^49,75^ and will require repeated calibration. In both kinds of detection, it is worthwhile verifying if the calibration curve remains unaffected before and after a local pH measurement.^75^ For single-point local pH measurements carried out for short times, this may not be a critical issue. However, when the pH probe is used for long-term monitoring or for imaging purposes, it must be ensured that no significant calibration errors are introduced due to drift or instabilities. In this context, it must be ensured that the leaching of pH-sensitive probes or the dissolution of metal oxide layers or ionophore components is suppressed.

When using voltammetric detection, it is possible to choose between an increased pH resolution and a large dynamic range. For the former case, it would be sufficient to use a redox probe (monomer or polymer) that works in a small pH range but is quite sensitive so that the smallest change in pH can be detected. A pH resolution of 0.02 pH units could be achieved for a polyaniline modified nanocapillary tip,^98^ while a resolution approaching 0.01 pH units could be achieved using a nanocapillary tip filled with a zwitterionic membrane.^37^ In pH sensing applications discussed here, we would like to measure local changes close to the surface and on most occasions in a buffer solution. Since we are often in the steady state regime,^95^ the dynamic range in most cases can be low.^37^

### In Operando and in Situ Sensing

Although electroanalytical strategies have several advantages over marker-based optical methods, one key issue is that the presence of an electrode close to the investigated interface disturbs the diffusion profile of the chemical species involved in the reaction/process. When using electrodes of diameter in the micrometer range, we are able to extract local pH information only several micrometers away from the interface. In order to observe pH changes closer to the surface, NEs have been successfully used to obtain local pH information, e.g. at a distance less than 100 nm from the interface.^37,49,76^ RRDEs provide a key advantage here, since the pH measurement at the ring electrode does not introduce mass-transport limitations for the reactions at the disk electrode.^4,75,95,147^ When studying biointerfaces in situ, it is also important that the sensor is highly selective to pH. Here, complex media are used, and matrix effects need to be considered when operating in such environments.

### Time Resolution

One key challenge for all discussed local pH sensing applications is time resolution, which depends on two limiting factors. The first one concerns the measurement resolution, i.e., how quickly the electrochemical method can detect a reliable signal. In the case of voltammetric detection, the use of appropriate NEs should in principle allow for fast scan rates. Indeed using a membrane-modified nanopore, a time resolution in the millisecond range could be demonstrated.^37^ With potentiometric sensors, the high impedance of the membrane results in a large RC time constant, which limits the resolution that can be achieved. A second aspect is the response time of the active layer containing the pH sensitive species. For potentiometric sensors, this is defined by the time the species needs to reach equilibrium. A long response time would substantially prolong pH mapping experiments, which is a fundamental challenge. Another central hurdle specific to multiprobe electrodes is crosstalk between the different electrodes on the same tip.

### Long-Term Operation

One of the important goals of local pH sensing is to follow the evolution of the pH during the occurrence of an interfacial process. Depending on the application, the duration of the interfacial phenomenon can be very long, e.g. a few hours when studying biointerfaces.^99^ For imaging purposes, it is also necessary that the electrode be stable for the entire duration of the scan. Therefore, the capability of long-term operation is a critical issue for local pH sensors. The stability is strongly influenced by the experimental conditions and the nature of the active sensing layer. Harsh conditions such as organic solvents, strong acids and bases, high temperature, or interfering ions can drastically affect the lifetime of the sensing electrodes. Ionophore-based electrodes have demonstrated capability for continuous operation up to several days.^52^ For electrodes modified with redox species, it has to be ensured that the attached molecules or layers are stable enough during the entire measurement duration. One way to prove the sensing performance is to perform sensor calibration before and after the experiment.

### Performance Comparison

When analyzing the performance, it turns out that it is difficult to directly compare the performance of different kinds of sensors. Here, there is a need to identify some standard conventions in reporting methods to measure sensitivity and response time by properly considering drifts. There have been some attempts to standardize the characterization of pH response in bulk sensors;^167^ however, this has not yet been discussed for UMEs or for local pH sensing. Despite this difficulty, we tried to collect the key performance aspects of reported local pH sensors in Table 1. As the temporal resolution is not considered separately for the sensor components, only the specified temporal resolution of the overall system is discussed, which is listed here under response time.

**Table 1:** Table Showing Key Parameters of the Reported Local pH Sensors^a^

Type	System	Refs	Electrode Diameter [μm]	pH Range	Slope [mV/pH]	Response Time [s]	Case Study (*Ec/Cn/Bio*)
Ionophore	Hydrogen/Proton Ionophore I	(132)	2	5–12.5	57.1	-	*Cn*
		(52)	20	4–12	59	-	*Ec*
		(41), *sc*	25	4–10	59	<0.5	*Bio*
		(54)	250	2–12	55.2	-	*Cn*
	Hydrogen/Proton Ionophore II	(55)	0.150	6.2–7.7	54–57	-	*--*
		(53)	5	3–9	49–59	20	*Cn*
		(141)	10	3–10	57	6	*Cn*
		(51)	60	2–10	55	1	*Cn, Bio*
	LIX	(123, 124, 163)	10	4–10	55–56	-	*Bio*
	Hydrogel (GOx/PLL)	(37)	0.050	4–9	*15–20	0.02	*Bio*
Metal Oxide (MOx)	Au/IrOx	(72)	25	3–7	61	1	*Bio*
		(74)	25	4–9	70	-	*Bio*
		(125)	25	4–9	62–64	-	*Ec*
		(145)	25	2–11	59–90	-	*Ec*
	Pt/IrOx	(161)	12.5	2–12	63.4	1	*Cn*
		(71)	25	3–7	71	3	*Bio*
	C/IrOx	(86)	5	1–12	58–63	30	*Bio*
		(76)	11.8	2–10	79	-	*Cn*
	Ir/IrOx-NPs	(83)	10	2–12	64	-	*Ec, Cn*
	Sb/Sb_2_O_3_	(80)	5	3–11	52	-	*Cn*
		(82)	15	3–11	46	-	*Cn*
		(78)	15	3–11	42	-	*Cn*
	RRDE: Au/IrOx	(73)		2–13	58	-	*Ec*
		(147)		2–11	56.9	3.7	*Ec*
		(75)		2–12	77	10	*Ec*
	RRDE: Pt/IrOx	(4)		4–10	74	-	*Ec*
Monomer	C/Syringealdazine	(49)	0.050	2–12	*54	1.2	*Ec*
		(91)	37	5–8	*60	<45	*Bio*
	Au/4-HATP	(50, 126, 130)	50	2–10	*57	5	*Ec*
	RRDE: Au/4-HATP	(95, 150)		4–13	v61	-	*Ec*
Polymer	Au/PANI	(98)	0.340	2–12	56	2	*Cn*
	Pt/PANI	(99)	25	4–8	53	<10	*Bio*
Metal	Au (Au_2_O_3_ reduction)	(109)	10	0–13	58–63	18	*Ec*
	Pt (CV)	(5)		10–13	*59	-	*Ec*
	Pt (PtO reduction)	(116)	25	12–14	*30–70	-	*Ec*
	Pt (HER)	(117)	0.600	1–8	*56	-	*Ec*

a For the electrode diameter, the smallest reported diameter is listed. For reasons of clarity, the errors in slope estimation are omitted in the table. Slope values were extracted from potentiometry, except those marked with a *, where voltammetry was applied. *Ec*: Electrocatalysis. *Cn*: Corrosion. *Bio*: Biointerfaces. *sc*: solid-contact electrode. LIX: Liquid-ion-exchange membrane (the exact composition was not reported here). GOx: Glucose oxidase. PLL: Poly(lysine). NPs: nanoparticles.

## Conclusions and Outlook

In summary, we have presented the progress that has been made in the development of sensors for detecting pH locally at or close to surfaces. We identified the key detection principles and discussed the advantages and drawbacks of the different electroanalytical readout strategies. Potentiometry using membrane-based electrodes has long been the classical method to detect pH. Several potentiometric pH sensors have been very successful in estimating the local pH at various interfaces. The results collected in this review demonstrate that there is significant potential in the use of voltammetric methods, for which electrodes modified with pH-sensitive probes are ideally suited. The use of metallic electrodes without any modification is another viable approach in selected buffers. Since miniaturization of such electrodes is rather straightforward, this methodology appears to be very promising for mapping local pH information with a good spatial resolution. A central challenge for pH mapping is the need for high temporal resolution. Also here, the voltammetric methods may be expected to deliver an improvement e.g. by utilizing pulsed potential or fast scan methods.^118^ Moreover, voltammetric methods are comparatively less susceptible to interference, and hence, the pH calibration is more reliable. Most of the reported applications of local pH sensing are model system studies, where the potential of local pH sensing becomes evident.

In the future, with the improved development of the reported probing methods, it can be expected that this analytical tool may provide new information when its application is extended to other interface-related systems and problems. There is much scope for optimizing the electrodes used for local pH sensing. For example, new methods to reduce the UME or NE diameter may help to improve the spatial resolution. The use of ionophores and redox species for local pH sensing is still in its infancy. Mostly standard and commercially available ionophores have been used to date. By exploiting the vast knowledge in the field of ionophores and ion-selective electrodes, it is expected that local pH sensing using potentiometric sensors will receive a boost. Apart from the redox species reported to date, there are a large number of other redox species that exhibit a pH-dependent formal potential. Moreover, the potential of polymeric redox probes for local pH sensing has not yet been fully exploited. In the future, it can be expected that other candidates will be investigated for improved stability and selectivity. From an instrumental perspective, it is expected that novel electroanalytical techniques (e.g., pulse-based methods) will provide some improvement in time resolution and the sensing performance. Finally, simultaneous mapping of the relevant chemical species together with pH mapping might be essential to obtaining a complete picture in mechanistic studies. Multiprobe electrodes may prove to be crucial in such kinds of measurements.

## Vocabulary

Two-electrode sensing configuration: an electrochemical cell, consisting of a sensing or indicator electrode and a reference electrode. In order that the potential of the reference electrode remains unchanged, the amount of current flowing through the cell must be very low (typically in the sub-nA range).
Electroanalytical techniques: analytical methods that use the measurement of a potential, current, or charge in an electrochemical cell to quantify the activity or concentration of an analyte species.
Potentiometric method: electroanalytical technique in which the potential is measured between a sensing electrode (also called an indicator electrode) and a reference electrode. The potential is measured in a static condition; i.e., the current passing through the cell is negligible.
Voltammetric method: electroanalytical technique in which the current is measured as a function of a time-varying potential applied to the working electrode.
Membrane potential: (in electroanalysis) the difference in potential developing across a membrane which separates two different phases. Typically, the activity of the chemical species is different in the two phases.
Proton ionophore: molecule that selectively binds protons.
Electrified interface: interface between a solid electrode and a solution with which the electrode is brought into contact. In addition, the potential at the electrode is actively controlled with respect to a reference electrode in the solution.
